# Notices

**Published:** 1867-11

**Authors:** 


### *Notices.*

The New-York Medical College for Women will begin their Fifth Annual Term of twenty weeks at the College in Twelfth street, two doors east of Fourth Avenue, the first Monday in November. Address the Dean, Mrs. C. S. Lozier, M. D., 361 West, 34 st., New-York, or the Secretary, Mrs. C. F. Wells, care of Fowler and Wells. We have been requested to give the above notice, and being of an obliging turn of mind naturally, we do so. We know of no valid reason why men should monopolize the practice of medicine; but there is to our mind a grave difficulty in the way of Lady-Doctors. Who is to take care of my baby while my wife is absent in recovering John Smith from an attack of delirium tremens, calling on her way home to administer an emetic to Tom Brown who is agonizing from the effects of an over supply of lobster salad, washed down with champaign? Is the life and limb of my own little cherub to be entrusted to the care of an ignorant and unprincipled hireling, while its mother has gone out to earn a dollar or two by looking after the health and happiness of "the gluttinous man and the publican?" If the Woman's College would grant diplomas to those ladies only who are so ugly it would be impossible for any man to woo or wed, then we withdraw our objection in part, not altogether, because as a general rule perhaps ugly women maket he best and most faithful and conscientious wives, while "perfect beauties" make no wives at all, no more than it is possible for a very handsome man to make a good husband. It does seem to us that the true sphere of women is motherhood, to sanctify home and to imbue the tender mind of her offspring with all that is beautiful in truth, pure in sentiment, and holy in religion, while she herself should be relieved from the drudgeries of life by the services of those of her sex who remain unmarried or are in widowhood. A true woman, a faithful mother is but a little lower than the angels; all honor and deference to such while living, and revered be their memory when they have passed away.

Gymnastics. Dr. Martin will reopen his Private Classes in Light or Musical Gymnastics in the hall of Harvard Rooms, corner Sixth Avenue, and 42d street, October 3d, 1867. He is a skilful, enthusiastic and conscientious instructor in those exercises which promote exactness, symmetry and strength of body; elegance, ease and endurance of action, quickness, flexibility and resonance of will and physical energy.

